# Reply

**DOI:** 10.1016/j.jcmgh.2022.07.004

**Published:** 2022-08-06

**Authors:** Heikki Virtanen, Daniel Garton, Jaan-Olle Andressoo

**Affiliations:** Department of Pharmacology, Faculty of Medicine and Helsinki Institute of Life Science, University of Helsinki, Helsinki, Finland; Department of Pharmacology, Faculty of Medicine and Helsinki Institute of Life Science, University of Helsinki, Helsinki, Finland; Department of Neurobiology, Care Sciences and Society, Neurogeriatrik, Karolinska Institutet, Sweden

We recently published an article in *Cellular and Molecular Gastroenterology and Hepatology* titled “Myenteric neurons do not replicate in small intestine under normal physiological conditions in adult mouse,”^1^ in which we found no evidence for DNA replication occurring in about 70% of adult enteric myenteric neurons in 1 week as reported by Kulkarni et al.^2^ in 2017.

In response to our article, Drs. Kulkarni and Pasricha submitted a “Letter to the Editor” to *Cellular and Molecular Gastroenterology and Hepatology* titled “Detecting adult enteric neurogenesis in the context of adult ENS homeostasis,” where the authors make 2 very important claims. First, Drs. Kulkarni and Pasricha assert that their results from 2017 have been independently validated. This is an important argument, because in science independent verification of results is the sole criteria for reproducibility, making observations into facts. Second, the authors propose that our methodology was inappropriate to detect DNA replication specifically in the enteric ganglia.

Regarding the first issue, Drs. Kulkarni and Pasricha write that “The nature and the ability of adult enteric [neural precursor cells] to cycle at steady state conditions was independently validated” and cite De Vadder et al.^3^

However, we note that De Vadder et al. report that in animals with a normal microbiome less than 1% of Nestin-positive cells in colonic myenteric ganglia express Ki67, a marker of cellular proliferation.^4^ De Vadder et al.^3^ used Nestin as a proxy for neuronal precursors but the neuronal identity of Ki67/Nestin-double-positive cells was not verified. Thus, we cannot be certain if these rare Ki67/Nestin-double-positive cells (<1%) were indeed neurons, because multiple cell types in the gut express Nestin.^5^ Regardless of this important issue, the De Vadder et al.^3^ study does not reproduce the results reported by Kulkarni et al.^2^ that about 70% of enteric neurons go through DNA replication every week.

Second, Drs. Kulkarni and Pasricha in their letter propose that overnight fixation in 4% paraformaldehyde overfixes the tissue, because they had used fixation for 1 hour in most of their experiments. They suggest that overnight fixation, although not affecting the ability to detect proliferating cells in the epithelium where we document the expected rate and location of proliferating cells in the crypt, somehow affects the ability to detect the thymidine analogues we used to label cells in S phase. To support their claim, Drs. Kulkarni and Pasricha argue that enteric neurons are surrounded by a blood myenteric barrier,^6^ which may prevent access of the antibodies after overnight fixation.

There are several reasons why this idea cannot explain our divergent findings:

1. The microtome blade cuts nuclei and cell bodies at random, exposing the interiors. The blood myenteric barrier around the cells is thus irrelevant.
2. We observe excellent staining of enteric neurons using antibodies against the enteric neuron marker protein HuD; thus antibodies do diffuse well into enteric neurons even after overnight fixation.
3. To avoid potential immunohistochemistry-related methodologic hurdles, we repeated experiments with EdU and “click chemistry” detection using AlexaFluor 594 azide with a molecular weight ∼0.8 kDa, which is about 190 times smaller than antibodies (∼150 kDa), and thus cannot be diffusion limited.
4. A clear DNA replication signal was observed also inside the enteric ganglia of our images (see for example Figure 2Q-R).^1^ Positive cells inside the ganglia, however, were not neurons.
5. We observe DNA replication-positive cells not only in the epithelium but also in the lamina propria, similar to what has been reported by other studies,^7^^,^^8^ and the overall number of EdU and IdU positive cells does not substantially differ excluding differences in diffusion rates caused by cross-linking.

Next, Drs. Kulkarni and Pasricha argue that: “…their failure to detect thymidine analogues in any cell within the myenteric ganglia and especially in myenteric glial cells that also cycle at steady state^9^^,^^10^ suggests that their methods may not have been optimized.” This statement is incorrect. As noted previously and shown in the figures of our paper, we document DNA replication-positive cells in the ganglia and in other regions of the gut including in the lamina propria, muscle layer, and in the epithelium. Analysis of glial proliferation was not our objective, so we did not co-stain for glial markers.

Regarding the issue of glial proliferation “at steady state,” we note that the papers cited by Drs. Kulkarni and Pasricha do not quantify myenteric glia proliferation.^9^^,^^10^ Analysis of scRNAseq data by Zeisel et al.^10^ indicated that there is a population of glial cells that may proliferate based on gene expression pattern. However, Zeisel et al.^10^ only analyzed developing animals and do not directly demonstrate glial proliferation. Furthermore, current literature suggests that unless the gut is damaged, adult glia proliferates at a very slow rate. Joseph et al.^11^ found only 2.8% of enteric glial cells to be positive for BrdU after BrdU was administered for 6 weeks followed by a 6-week chase. Thus, based on current research enteric glial proliferation in the healthy gut is a rare event.

Finally, Drs. Kulkarni and Pasricha discuss apoptosis in enteric neurons and propose that their result of high levels of apoptosis has also been observed by others. Apoptosis in the adult ENS was not our topic of study. However, it is important to cite all, at least technically well performed, papers on the topic. Several studies have searched for apoptosis in the ENS using various tools and observed no evidence for apoptosis or cleaved caspases during late embryonic development^12^^,^^13^ or in the adult gut.^14^ The study cited by Drs. Kulkarni and Pasricha^15^ indeed reports that about 10% of enteric neurons are positive for cleaved Caspase 3 in the adult gut. However, this study^15^ may be confounded by sampling errors, because the authors only counted 4 enteric ganglia per animal and defining healthy gut apoptosis levels was not their main objective.

In conclusion, we thank the editors of *Cellular and Molecular Gastroenterology and Hepatology* for the opportunity to respond and to Drs. Kulkarni and Pasricha for an interesting discussion. In science, it is very hard to prove that something is not there if something has been claimed to be there. Should new facts emerge that show that most enteric neurons do turn over in 1 week despite our work and prior art from 7 other studies, we are happy to change our minds at that moment. Until then, we trust our study and the work of our colleagues, which altogether find that there is little DNA replication in adult enteric neurons in a healthy gut.
